# Machine Learning-Based Microclimate Model for Indoor Air Temperature and Relative Humidity Prediction in a Swine Building

**DOI:** 10.3390/ani11010222

**Published:** 2021-01-18

**Authors:** Elanchezhian Arulmozhi, Jayanta Kumar Basak, Thavisack Sihalath, Jaesung Park, Hyeon Tae Kim, Byeong Eun Moon

**Affiliations:** Department of Bio-Systems Engineering, Institute of Smart Farm, Gyeongsang National University, Jinju 52828, Korea; mohanachezhian@yahoo.com (E.A.); basak.jkb@gmail.com (J.K.B.); max7set@gmail.com (T.S.); jaesung.park@gnu.ac.kr (J.P.); bioani@gnu.ac.kr (H.T.K.)

**Keywords:** indoor air temperature, indoor relative humidity, swine building microclimate, ML models, smart farming

## Abstract

**Simple Summary:**

Indoor air temperature (IAT) and indoor relative humidity (IRH) are the prominent microclimatic variables. Among other livestock animals, pigs are more sensitive to environmental equilibrium; a lack of favorable environment in barns affects the productivity parameters such as voluntary feed intake, feed conversion, heat stress, etc. Machine learning (ML) based prediction models are utilized for solving various nonlinear problems in the current decade. Meanwhile, multiple linear regression (MLR), multilayered perceptron (MLP), random forest regression (RFR), decision tree regression (DTR), and support vector regression (SVR) models were utilized for the prediction. Typically, most of the available IAT and IRH models are limited to feed the animal biological data as the input. Since the biological factors of the internal animals are challenging to acquire, this study used accessible factors such as external environmental data to simulate the models. Three different input datasets named S1 (weather station parameters), S2 (weather station parameters and indoor attributes), and S3 (Highly correlated values) were used to assess the models. From the results, RFR models performed better results in both IAT (R^2^ = 0.9913; RMSE = 0.476; MAE = 0.3535) and IRH (R^2^ = 0.9594; RMSE = 2.429; MAE = 1.47) prediction with S3 input datasets. In addition, it has been proven that selecting the right features from the given input data builds supportive conditions under which the expected results are available.

**Abstract:**

Indoor air temperature (IAT) and indoor relative humidity (IRH) are the prominent microclimatic variables; still, potential contributors that influence the homeostasis of livestock animals reared in closed barns. Further, predicting IAT and IRH encourages farmers to think ahead actively and to prepare the optimum solutions. Therefore, the primary objective of the current literature is to build and investigate extensive performance analysis between popular ML models in practice used for IAT and IRH predictions. Meanwhile, multiple linear regression (MLR), multilayered perceptron (MLP), random forest regression (RFR), decision tree regression (DTR), and support vector regression (SVR) models were utilized for the prediction. This study used accessible factors such as external environmental data to simulate the models. In addition, three different input datasets named S1, S2, and S3 were used to assess the models. From the results, RFR models performed better results in both IAT (R^2^ = 0.9913; RMSE = 0.476; MAE = 0.3535) and IRH (R^2^ = 0.9594; RMSE = 2.429; MAE = 1.47) prediction among other models particularly with S3 input datasets. In addition, it has been proven that selecting the right features from the given input data builds supportive conditions under which the expected results are available. Overall, the current study demonstrates a better model among other models to predict IAT and IRH of a naturally ventilated swine building containing animals with fewer input attributes.

## 1. Introduction

### 1.1. Research Significance

Climate change has intensified the impacts against agriculture production over the past few decades that makes bewilderment on the livelihoods of farmers and consumers. In the current scenario, producing high quality agricultural products using traditional farming methodologies is becoming arduous for the farmers. In 2030, the world would have to feed more than 8 billion people, whereas maintaining sustainable farming methodologies is an enormous challenge for food security [1]. Economic experts estimate the demand for milk and meat by 2050 could increase by 70 to 80% over current market demand [2]. However, extreme weather conditions directly affect the livestock sector in several ways, such as productivity losses, biological changes, and welfare issues [2]. There is a demand to adopt modern farming methods such as smart livestock farming (SLF), which are alternatives to conventional farming methods to address these challenges. SLF can provide optimal control strategy with the help of inexpensive and improved sensors availability, actuators and microprocessors, high performance computational software, cloud-based ICT systems, and big data analytics. The significance of well-managed animal welfare is not narrow to ethical aspects; it is vital to realize an effective action of provoking animal commodities.

Maintaining a favorable environment in livestock building would assist in producing qualitative and healthier outcomes. The preeminent intention of adopting the SLF is to regulate the indoor microclimatic parameters like temperature and humidity at the optimum level [3]. The characteristics of indoor microclimate immensely influence the livestock production aspects such as animal health and welfare. The pigs are more sensitive to indoor climatic parameters than all other livestock, so that a constant temperature and humidity are the essential factors for their routine activities. In general, 16–25 °C of indoor temperature and 60–80% of indoor humidity are considered the optimal environment for pigs; such an environment is called a thermo-neutral zone (TNZ) [4,5]. The TNZ provides the welfare of animals, resulting in enhancing the voluntary feed intake and minimizing thermal and other environmental stress [6]. Maintaining proper temperature and relative humidity within the pig’s TNZ is the primary function of a microclimate controlling system [7]. Modelling the microclimate of livestock building by using outdoor parameters helps to regularize the indoor environment condition; moreover, it may guide the preparation of precautions from extreme outdoor conditions.

The indoor microclimate dynamics are majorly affected by the outdoor disturbance generated from either seasonal or daily meteorological changes being outdoor temperature variations, humidity changes, rainfall fluctuation, etc. Advanced microclimate models are vital to make microclimate controllers as smart, which may also act as supplementary to boost the controllers’ strategy. Heretofore researchers developed several models as dynamic, steady-state models, heat balance equations, computational fluid dynamics to predict indoor air temperature (IAT), and indoor relative humidity (IRH). Most of the previous models were developed by using the theoretical relationship between heat and mass transfer functions, energy-oriented facets, and indoor fluid dynamics [3,8,9,10]. Such mechanisms require complex information such as airflow dynamics, animal information, and fan specifications to derive the equations. Nevertheless, such kinds of models are limited to quality, quantity, missing values of data while predicting the naturally ventilated building’s IAT and IRH. Collecting attributes of those variables mentioned earlier are convoluted; thus, adopting advanced modeling techniques like artificial intelligence (AI) is key to simulate the microclimate in easier way.

Machine learning (ML) is a subdivision of AI, has reinterpreted the world in diverse forecasting fields for the past two decades. The rapid advancement of graphics rendering and computer synchronization combined is the reason for the excessive growth of ML popularity than other prediction methods [11]. The ML models are capable of adaptive learning from the data, and it can improve themselves from subsequent training, trends, and pattern identification. Such inherent characteristics have driven them to handle complex investigations effectively. Applying such technologies could analyze the large data sets more effectively with relative ease than physical or statistical models. Especially for determining linear and nonlinear variables that follow time-series such as indoor microclimate modelling field, the ML-based models have proven it outperformed the statistical models [10]. Several training algorithms are available for the ML framework, including linear regression (LR), decision trees regression (DTR), random forest regression (RFR), support vector regression (SVR), etc., have been developed to handle the regression and classification problems. Previous studies utilized artificial neural network (ANN) and ML models to predict the variables related to animal studies. For instance, ref. [10] utilized an ANN model to predict a swine building’s temperature and relative humidity, whereas the growth performance of swine was analyzed with decision trees and support vector machines by a previous study [12]. Likewise, ref. [13] predicted the skin temperature of pigs based on the indoor parameters using an MLP. A previous study [14] employed MLP and classification and regression trees (CART) algorithms to predict piglets’ core, skin, and hair-coat temperatures.

### 1.2. Research Objectives

Through achieved significant certainty, ML models have been utilized to work out disputes such as prediction, classification, clustering, etc. Nevertheless, there is a knowledge gap in utilizing advanced modelling techniques to simulate the microclimate of a livestock building [15]. The current study tries to evaluate the performance of usual ML models while simulating the IAT and IRH of a swine building. 

Research on the depth and breadth of the applications and the state of the art of ML-based predictions of IAT and IRH of pig barn containing animals are scarce. Several models have been successfully developed and implemented to predict microclimate of other smart farm buildings like a greenhouse and plant factory. Like the other smart farm, ML models could be adopted in order to regulate the microclimate of pig buildings after optimization and calibration. Previous researchers mostly develop a single model and simulate the attributes and validation; therefore, the model’s robustness becomes a dispute. For instance, Ref. [10] employed a multilayered perceptron with a backpropagation model to predict the IAT and IRH of a swine building, and evaluate the model without comparison with other models. In contrast, refs. [16,17] simulates the indoor microclimate using the autoregressive integrated moving average (ARIMA) model. A comprehensive comparison and analysis between the other popular model performances lack such literature; those studies build a model and validate it quickly. Therefore, the leading intention of the current study is to build and investigate extensive performance analysis between popular ML models in practice used for IAT and IRH predictions. 

Typically, indoor climatic parameters of any animal buildings are dependent variables that are subject to significant change by the external environmental parameters. Unlike mechanically ventilated buildings, naturally ventilated swine buildings indoor IAT and IRH aerodynamics eminently vulnerable to outdoor climate and biological factors of the animals present [18]. The outdoor climate data is accessible and ubiquitous, whereas collecting biological data involves skin temperature, behavioral changes, health aspects, etc. It is not limited to predicament data acquisition; it also affects the physical equilibrium and homeostasis of the animals while collecting data [19]. Considering the above factors, the current research used accessible factors such as external environmental data without considering the biological factor of the animals to simulate the models.

Determination of input data is the bottom line of any modelling criteria yet crucial consideration in diagnosing the exquisite functional form of ML models. Choosing the right input variables involves improving the accuracy of the algorithm; also, it dominates the calculation speed, training time, training complexity, comprehensibility, and computational effort of the simulation [20,21,22]. The present study analyzes the performance of the models with feature-selected datasets and available datasets; it also suggests the optimal input selection to feed the models from the available datasets.

## 2. Materials and Methods

### 2.1. Arrangement of Swine Building

**Figure 1:**
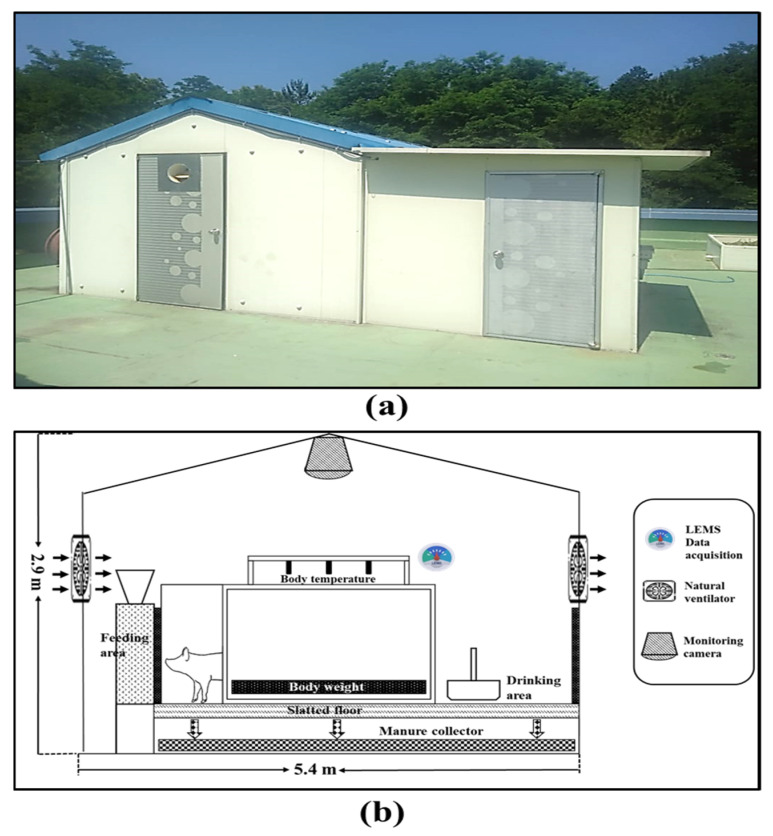
(**a**) Outdoor view of the model swine building which was used for current experiment; (**b**) sensor placement and indoor schematic of model swine building.

The current study was conducted at a model swine building located in Gyeongsang National University, Jinju-Si, the Republic of Korea, with 2.9 m width × 5.4 m length × 0.05 m thick roofs as shown in Figure 1. The GPS coordinates for the site was 35°09′6.26″ N, 128°05′43.838″ E [23]. The heat conduction is diminished by over 40% while utilizing slatted floors compared to the concrete floors in a naturally ventilated pig barn [24]. The model swine building used polypropylene copolymer slatted floors to decrease heat transmission, and the total area of the barn was 13.26 m^2^ (1.32 m^2^/pig). Ten crossbreeds (American Yorkshire × Duroc) pigs with an average body weight of 86.4–142.4 kg were grown in the model swine building throughout the experimental time. The trial building incorporates an automatic infrared sensor-based feeder (robust military automatic feed system, South Korea) integrated with the body weight and body temperature estimation scales. The pigs were offered nutritionally balanced dry feed to meet apparent digestible energy (DE) 3500 kcal/kg twice a day (09:00 h and 17:00 h). The pigs were provided 1.5–3.2 kg/day/pig of dry feed, as suggested by the Institutional Animal Care and Use Committee (IACUC) of Gyeongsang national university during the overall experimental time.

### 2.2. Sensor Data

**Figure 2:**
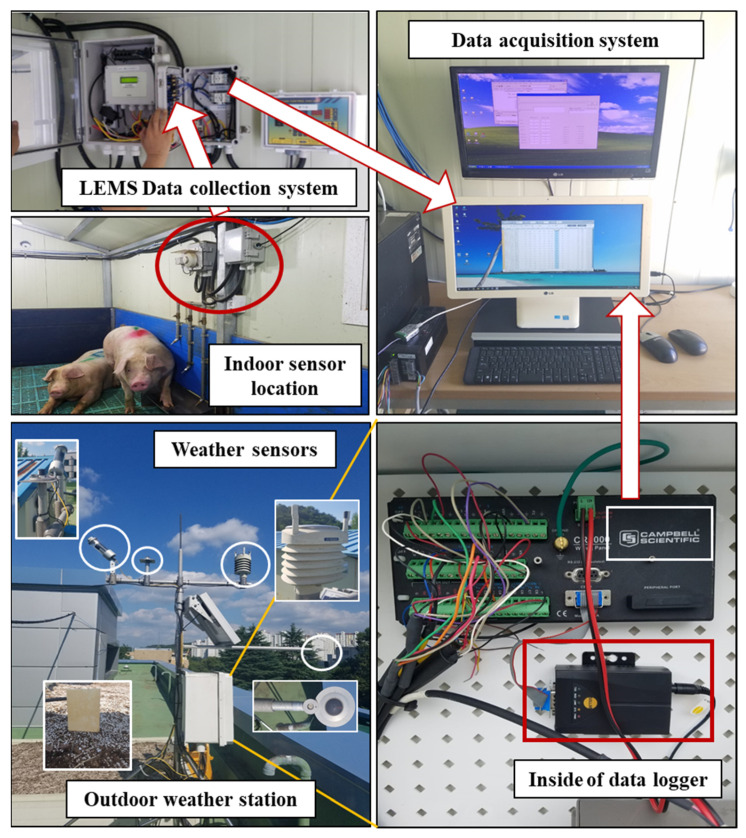
Devices used for data acquisition of indoor (LEMS) and outdoor (Campbell scientific weather station) including sensor extensions; data transmission process during LEMS and CR1000X data storage to the primary database.

A research-grade weather station (model: MetPRO, Producer: Campbell Scientific, Logan, UT, USA) was installed at 26 m away from the model swine building to collect the outdoor climatic variable, used as a predictor/independent variables. A digital air temperature and humidity sensor (CS215-L), a wind sentry set with an anemometer (03002-L), rain gage with a 6-inch orifice (TE525-L), pyranometer (CS300-L), barometric pressure Sensor (CS100), and reflectometer (CS655), such customized sensors were comprised to the weather station for the data reception. A data logger (model: CR1000X), which is capable of storing the data from the sensors and parallel transportation of data to the computer, was annexed to the weather station. Indoor microclimatic parameters were recorded by utilizing a livestock environment management system (LEMS, AgriRoboTech Co., Ltd., Gyeonggi, South Korea), which is capable of acquiring data from inside of pig barn and store the accumulated data. The collected data considered as the response variable for the current study. However, the weather station and LEMS data were stored in the database management system for analysis purposes. The complete details of the sensor, sensor placement, and equipped devices are disclosed in Figure 1b and Figure 2 in a detailed manner.

For this study, each computerized sensor data was stored at 10-min intervals according to the experimental design from 17 September to 5 December 2019. During the experimental time, pigs were grown in the model swine building. Since the final goal of this research is to optimize the actuators, the model pig barn was considered as a prototype. Overall, 2 response variables and 10 predictor variables data were used for the analysis. The details of collected independent and dependent variables with unit, mean, minimum, maximum, and standard deviation (SD) are explained in Table 1. The indoor microclimate may have affected by the biological factors of the animals such as body temperature, water drinking, feed intake, etc. Since the primary objective of the study is modeling the indoor parameter by considering the outdoor parameters, the current research averts biological factors.

### 2.3. Approach

#### 2.3.1. Multiple Linear Regression Model

Multiple linear regression models (MLR) are commonly used empirical models to solve nonlinear problems. These models are also popular among the fields such as weather prediction, electricity load, energy consumption, heat transfer, business forecast, etc. [25,26,27,28]. Generally, regression models examine the relative influence of the independent variables or predictor variables on the dependent variables or response variables. MLR models are popular among the forecast because of their non-complex structures, calculation interpretability, and the ability to identify outliers or anomalies in given predictor variables. An MLR model can be expressed by the following equation [25,26,27,28],

(1) $${Y=a}_{0}{+a}_{1}X_{1}{+a}_{2}X_{2}{+\ldots +a}_{i}X_{i}+Ɛ$$

where Y is the response (output) variable; X is the predictor (independent) variable (from X_1_ to X_i_); a is the regression coefficient to predict Y (from a_1_ to a_i_); a_0_ is the intercept/constant of the model; and the Ɛ is the noise or random error of the model.

#### 2.3.2. Decision Tree Regression Model

**Figure 3:**
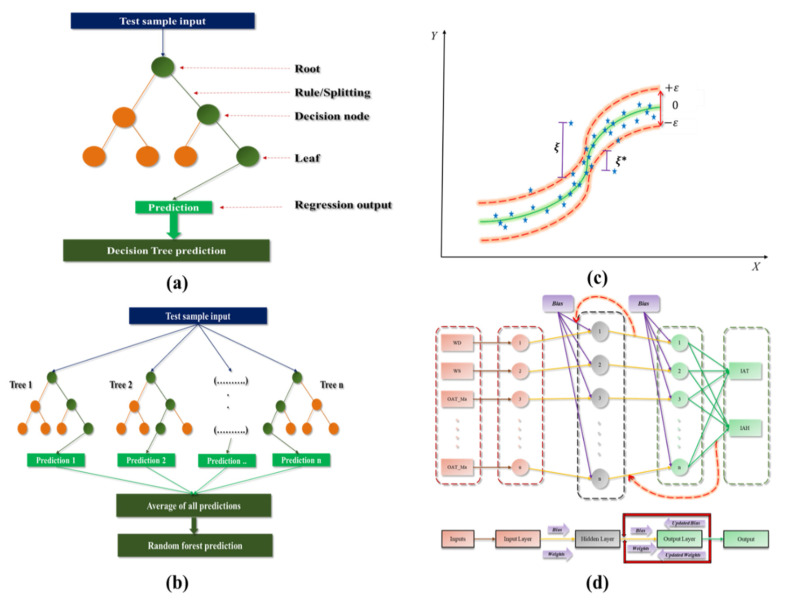
Important parameters and operational blue print of (**a**) Decision tree regression model, (**b**) Random forest regression model, (**c**) Support vector regression model, (**d**) Multilayered perceptron—Back propagation model.

Unlike other ML models that are considered as a black-box model while operation, decision tree regression (DTR) models are own opposite characteristics among the other models. Compared to the other supervised algorithms, DTR is popular for the self-explanatory/rule-based by nature; data interpretability for a response subject to the predictor variables could formulate visually [11]. DTR models were initially developed to solve the classification problem and manipulated to solve the classification and regression problem (CAR). The schematic diagram of the DTR model is shown in Figure 3a, where each node represents features, each branch of the tree represents a rule/decision, and each leaf of the tree represents regression values. The DTR models predict the output by calculating the probability of an outcome based on the feature influence. DTR uses the entropy function and information gain as the relevant metrics of each attribute to determine the desired output. Entropy/information entropy is used to measure the homogeneity of an arbitrary collection of samples. The information gain is applied to calculate the amount of an attribute, which contributes to estimating the classes. The entropy and information gain can be expressed by the following Equations (2) and (3) [11,29,30],

(2) $$H=-\overset{C_{T}}{\underset{c=1}{\sum }}p_{\mathrm{Ti}}{\cdot \log}_{2}\left(p_{\mathrm{Ti}}\right)$$

(3) $$\mathrm{Information}\;\mathrm{Gain}\;\left(X,T\right)=H\left(T\right)-\overset{n}{\underset{i=1}{\sum }}\frac{\left|T_{i}\right|}{\left|T\right|}\cdot H\left(T_{i}\right)$$

where p_Ti_ is the proportion of data points; C_T_ is the total number of classes; T_i_ is the one sample among all the n subsets in which the total amount of training data T was divided due to an attribute X.

#### 2.3.3. Random Forest Regression Model

The random forest (RF) algorithm is commonly known as an ensemble of randomized decision trees (DTs). RF algorithm has a similar operational method of DTs since RF lain on the same family of algorithms [14,31,32]. Consistently the use of DTs is uncertain since those are prone to overfitting, not accurate with large datasets, resulting in poor outputs for an unseen validation set. To mitigate the limitations of DTs, RF was deployed to determine the CAR interpretations more efficiently. Simply, RF is a collection of DTs where all the trees depend on a collection of random variables. However, RF models function as a “black box” since there is a limitation to observe each tree. Unlike DT, the interpretability of prediction is limited to visualization. In RFR, the output is predicted by averaging output of each ensemble tree. Subsequently, RFR produces a threshold for generalization error, which could be helpful to avoid overfitting. The generalization error of RFR is estimated by the error for training points, which are not contained in the bootstrap training sets (about one-third of the points are left out in each bootstrap training set), called out of bags (OOB) error. The process of OOB estimation is the reason behind their non-overfitting nature since OOB is indistinguishable from the N-fold cross validation. The RFR has the following essential characteristics [14,31,32],

- Selecting random features,
- Bootstrap sampling,
- OOB error estimation to overcome stability issues, and
- Full depth decision tree growing.

After all, the predictions of all trees are averaged to produce final predictions. The mathematical expression of RFR could be expressed as the following equation [14,31,32],

(4) $$Y=\frac{1}{M}\overset{M}{\underset{i=1}{\sum }}H\left(T_{i}\right)\;\mathrm{where}\;H\left(T_{i}\right)\;\mathrm{from}\;\mathrm{DTR}$$

where M is the total number of trees, Y is the final prediction; H (T_i_) is a sample in training set.

#### 2.3.4. Support Vector Regression Model

In 1992, Vapnik proposed a supervised algorithm named support vector machine (SVM), which was regarded as a generalized classifier [33]. Initially, the SVM algorithm was widely used to solve the classification problem in the name of support vector classification (SVC). Later Druker [33] extended it to solve the nonlinear regression problems with the name of support vector regression (SVR). A hyperplane that supported by a minimum margin line and a maximum margin line along with the support vectors were the conception elements of SVR [31,34,35]. The schematic diagram of the one-dimensional support vector regression used for regression showed in Figure 3. Let consider the available dataset with n samples, where x is the input vector, and y is the corresponding response variable of the dataset. The SVR generates a regression function to predict the y variable. This process can be expressed [31,33,34,35] by

(5) y=f(x)=ω·φ(x)+b

where x is the input of the datasets; ω and b are the parameter vectors; φ(x) is the mapping function, which is introduced by the SVR. In case of a multidimensional dataset, y can have unlimited prediction possibilities. So, a limitation for the tolerance introduce to solve the optimization problem [31,34,35], which could be expressed as

(6) $$\mathrm{Minimize}:\;\frac{1}{2}\;\left|\left|\omega^{2}\right|\right|+C\overset{n}{\underset{i=1}{\sum }}\left(\xi_{i}{+\xi }_{i}^{*}\right)\;\mathrm{Subject}\;\mathrm{to}\;\{\begin{array}{c} y_{i}-\left(\omega \cdot \varphi \left(x\right)+b\right)\leq Ɛ{+\xi }_{i}\\ \left(\omega \cdot \varphi \left(x\right)+b\right)-y_{i}\leq Ɛ{+\xi }_{i}^{*}\\ \xi_{i}{,\xi }_{i}^{*}\geq 0,\;i=1,\;\ldots ,\;n \end{array}$$

where ε is the minimum and maximum margin line/sensitivity zone of the hyperplane; ξ and ξ_i_* are the slack variables that measure the training errors which subjected to Ɛ; and C is the positive constant. The slack variables were utilized to minimize the error between the sensitive zones of hyperplane. The sensitive zones can also be expressed using Lagrange multipliers, the optimization techniques to solve the dual nonlinear problem can be rewritten as the following equation [31,34,35],

(7) $$\min:\;\frac{1}{2}\;\overset{n}{\underset{i=1}{\sum }}\overset{n}{\underset{j=1}{\sum }}\left(a_{i}-a_{i}^{*}\right)\left(a_{j}-a_{j}^{*}\right)K+Ɛ\overset{n}{\underset{i=1}{\sum }}\left(a_{i}{+a}_{i}^{*}\right)-\overset{n}{\underset{i=1}{\sum }}y_{i}\left(a_{i\;}-{\;a}_{i}^{*}\right)\;\mathrm{Subject}\;\mathrm{to}\;\{\begin{array}{c} \sum_{i=1}^{n}\left(a_{i}-a_{i}^{*}\right)=0\\ 0\leq a_{i},\;a_{i}^{*}\leq C,\;i=1,\;\ldots ,\;n \end{array}$$

where a_i_ and a_i_* are the Lagrange multipliers which subject to Ɛ; K is the kernel function. The kernel function use the kernel trick to solve the nonlinear problems using a liner classifier. Generally, linear, radial basis function (RBF), polynomial, and sigmoid are used kernel functions of SVR models [31,34,35]. The current study chose RBF as kernel function to optimize the SVR during the simulation after a random test of other kernel functions. The RBF kernel function can be expressed as the following equation,

(8) $$K\left(i,j\right)=\exp(-Ɣ{{|x}_{i}-x_{j}|}^{2})$$

where, Ɣ referred as the structural parameter of RBF kernel function. Finally, the decision function of SVR can be expressed as

(9) $$f\left(x_{i}\right)=\overset{n}{\underset{i=1}{\sum }}\left(a_{i}-a_{i}^{*}\right)K\left(x_{i}{,x}_{k}\right)+b$$

#### 2.3.5. Multilayered Perceptron—Backpropagation Model

Multilayered perceptron (MLP) along with the backpropagation (BP) technique is popular among ANN models [13,27,31]. Many researchers have proven and proposed that the MLP based model achieved dominant results in climate forecasting. The basic architecture of MLP is shown in Figure 3d. MLP is a feed-forward network with the three significant elements called the input layer as the first layer, hidden layer as the middle layer, and output layers as the final layer; each layer includes several neurons. The input layer represents the dimension of the input data and the hidden layer has n neurons, which is the fully connected network to the outputs (IAT and IRH). An MLP with three layers can be expressed mathematically by a linear combination of the transferred input metrics as [13,27,31]:

(10) $$y_{p}{=f}_{0}\left[\overset{n}{\underset{j=1}{\sum }}w_{\mathrm{kj}}f_{h}\left(\overset{m}{\underset{j=1}{\sum }}w_{\mathrm{ji}}x_{i}{+w}_{\mathrm{jb}}\right){+w}_{\mathrm{kb}}\right]$$

where y_p_ is the predicted output; f_0_ is the activation function for the output neuron; n is the number of output neurons; w_kj_ is the weight for the connecting neuron of hidden and output layers; f_h_ is the hidden neuron’s activation function; and m is the number of hidden neurons. w_ji_ is the weight for the connecting neuron of input and hidden layers; x_i_ is the input variable; w_jb_ is the bias for the hidden neuron; and w_kb_ is the bias for the output neuron [13,27,31].

The BP is a training technique, which again train every neuron with the updated weight and bias. This process involves in reducing the prediction error of the output layer. The updated weight can be expressed by the following expression

(11) $$W_{X}^{*}{=W}_{X}-a(\frac{\partial_{\mathrm{Error}}}{\partial W_{X}})$$

where W_X_* is the updated weight, W_X_ is the old weight, a is learning rate, ∂_Error_ is the derivative of error with respect to the weight. The error function for the BP training can be expressed as

(12) $$E=\overset{p}{\underset{p=1}{\sum }}E_{p}=\overset{p}{\underset{p=1}{\sum }}\cdot \overset{n}{\underset{k-1}{\sum }}{\left(y_{p}-y_{a}\right)}^{2}$$

where E is the error of the input patterns; E_p_ the square difference between the actual value and predicted value.

### 2.4. Choosing Input Datasets

**Figure 4:**
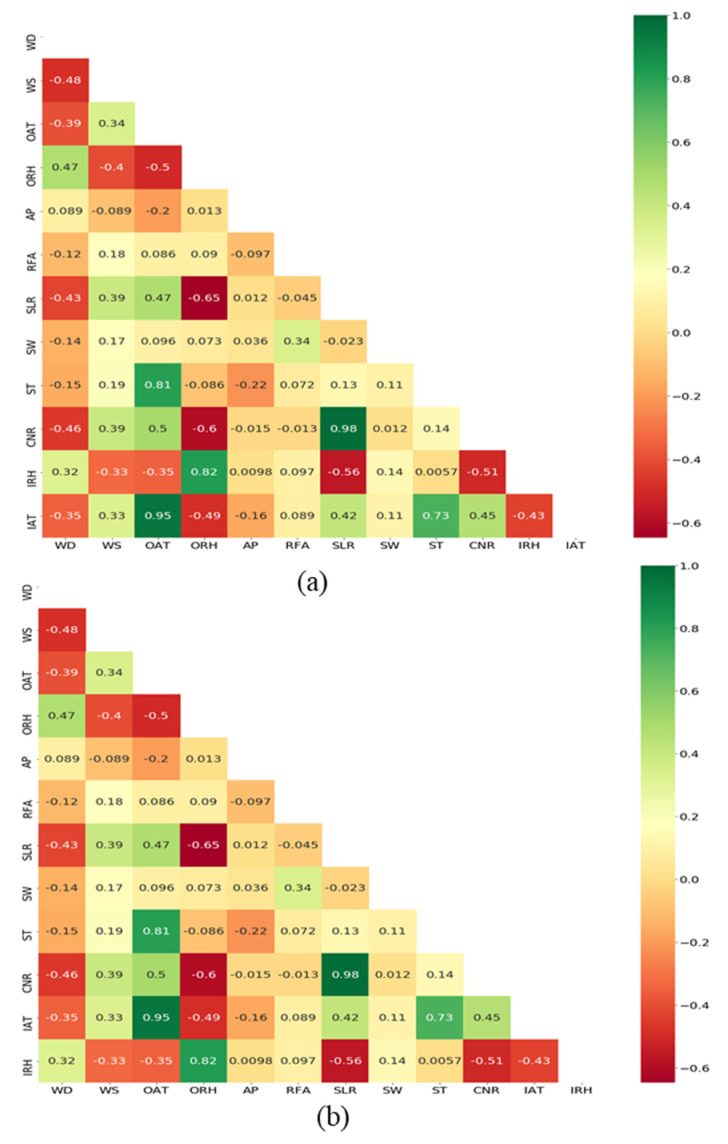
Heat correlation results between (**a**) Indoor air temperature (IAT) and (**b**) Indoor relative humidity (IRH) with other independent variables by using Spearman rank correlation coefficient approach.

As mentioned in the sensor data part, the outdoor and indoor variables were collected from the computerized sensors and it is explained along with mean ± standard deviation, standard error, and minimum and maximum values in Table 1. It has been reported that recording every meteorological parameter is complicated due to the unavailability or uncertainty of the sensor’s measurements. In this study, three different input datasets named S1, S2, and S3 were used to assess the models, which are illustrated in Table 2. To achieve the desired accuracy, it is essential to generate a reference for selecting the parameters that need to be recorded. The current study considers the use of different datasets as a useful method to ascertain the appropriate data that may have fewer variables and significant implications for predictions indeed [20,21,36,37]. So that the current study adopts the Spearman rank correlation coefficient approach in order to extract the best features, which is a commonly followed method to explore the relationships between attributes. Such correlation test aids to describe whether the relationship between independent and dependent factors are strong or not. Having a strong relationship, those independent attributes can be considered as a strong predictor of dependent attributes. The heat correlation results between IAT and IRH with other independent variables were showed in Figure 4. According to the rank correlation tests, the high correlated attributes were selected and used as dataset S3. The current study considers that ±0.5 as the high correlation value to choose as the S3 input set.

### 2.5. Assumptions for Modeling

**Figure 5:**
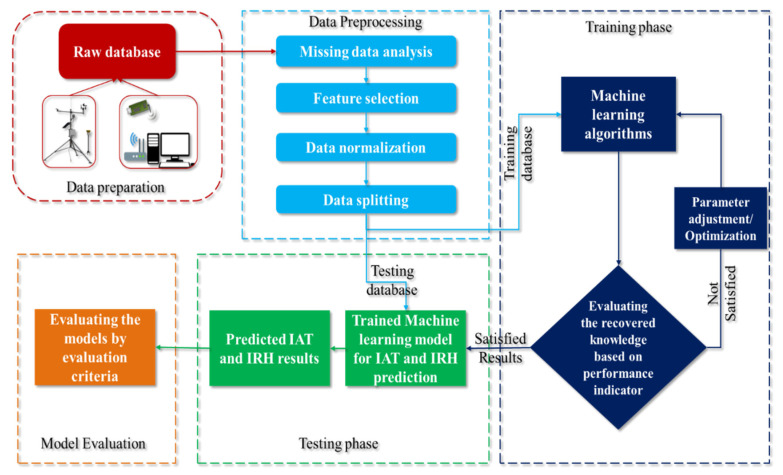
Phase by phase flow chart of the implementation of machine learning models for predicting IAT and IRH.

Throughout the aggregate workflow of this study has been explained systematically in Figure 5. As a first step, overall data sets were collected and stored at 10 min intervals from the sensors. At next, the stored data were subjected to the preprocessing methods as missing data analysis, feature extraction, data normalization and training and testing data partition. In collected datasets, there was no missing data/false data, so this research does not consider any techniques such as linear interpolation, k nearest neighbor algorithm, etc., for imputing the missing values [38]. The rank correlation test was used to select the right features from the available information, as mentioned in the input data part. A dataset with a different range of attributes used as input for any ML model will reduce the model’s learning efficiency and prediction capabilities. Since our attributes were in different ranges, the input data was mapped to a specific range to neglect the complications mentioned earlier. Minimum–maximum normalization is a popular preprocessing technique for ML modeling, which rescales the input features in the range of −1 to 1 or 0 to 1 [39,40]. The current study adopted the min-max normalization with the range between −1 (min) to 1 (max) to rescale the data, which could be expressed by the following equation [39,40,41],

(13) $$x_{\mathrm{nor}}=\frac{{2*(x-x}_{\min})}{{(x}_{\max}-x_{\min})}-1$$

where x_nor_ is the normalized data, x_max_ is the maximum of original data, x_min_ is the minimum of original data, and x is the original data. After the normalization applied to the input data, each attribute was changed to the −1 to 1 range. Though the ML models have been relatively efficient and popular in recent decades, training methods and the amount of feeding data have contributed to their success. More often researchers used 70:30 (training:validation), 80:20, or 90:10 partition to simulate the models [11,13,27,42,43]. The data partition scale for training and testing to be given during the simulation is assumed to be still unexplained and without any principled reason-based calculation. The current study utilized 80% of the data for training and 20% of data for testing. Hyper parameters such as learning rate, hidden layers, number of leaves, etc., are the key phenomenon, which may directly manipulate the behavior of any machine learning algorithms. Optimization/fine-tuning is a method to choose proper hyper parameters for desirable outcomes [14,31,44]. The current study adopted the grid search method to select the best parameters to model the machine learning algorithms. The range of tuned hyper parameters was shown in Table 3. The critical hyper parameters of all other ML models except MLR model were fine-tuned using the grid search method. In the next step, the abovementioned methodologies were followed before the training, and the training results were documented. During the testing phase, the IAT and IRH were predicted for 20% of untrained data sets using all ML algorithms. The results of both non-optimized and optimized models were documented to observe the performance of the models during the training and testing phase. At the final step, the model prediction results during the training and testing were evaluated by using mean absolute error (MAE), root mean square error (RMSE), and coefficient of determination (R^2^) methods, which could be expressed by the following equations [11,27,31],

(14) $$\mathrm{MAE}=\frac{\sum_{i=1}^{n}\left|y_{i}{-p}_{i}\right|}{n}$$

(15) $$\mathrm{RMSE}=\sqrt{\frac{\sum_{i=1}^{n}{{(y}_{i}{-p}_{i})}^{2}}{n}}$$

(16) $$R^{2}=1-\frac{\sum_{i=1}^{n}{\left(y_{i}-p_{i}\right)}^{2}}{\sum_{i=1}^{n}{\left(y_{i}-\frac{1}{n}\sum_{i=1}^{n}y_{i}\right)}^{2}}$$

All the ML models used for this study were developed using Python platform (Python version 3.7) and other statistical works were done with BM SPSS Statistics (version 26, IBM, Armonk, NY, USA).

## 3. Results

During the training phase and validation phase, the evaluation results were categorized by the input data type, model performance, and model comparison. In part named input datasets, the results obtained using S1, S2, and S3 datasets were deliberated. The performance of each model during the training and testing is illustrated in the model performance part. The percentage difference in all models’ results and the percentage difference between the models were discussed in the model comparison part.

### 3.1. Input Datasets

During the IAT predictions, the S3 dataset outperformed S2 and S1 during the testing phase. As mentioned above, in this part, the performance of models with three input data and the deviation percentage one among other datasets during the testing phase were assessed. All ML models outperformed when using S3 data. For instance, MLP obtained best performance (with S3) (R^2^ = 0.9913; RMSE = 0.4763; MAE = 0.3582) during the IAT’ testing predictions. Since the MLP performed better than other models during IAT’s testing results, it has been chosen for inter comparison between S1, S2, and S3. When compared to S2 and S1 results of MLP’s testing results, S3’s MAE was less by 5.2%, RMSE was less by 11.2%, and R^2^ was higher by 0.2%; when compared to S2 and S3 testing results, S3’s MAE was less by 26.13%, RMSE was less by 33.15%, and R^2^ was higher by 0.6%. Likewise, the MLR obtained the least performance among other models during the IAT’s testing prediction (R^2^ = 0.9354; RMSE = 1.332; MAE = 1.061). When compared to S2 and S1 testing results of MLP, S3’s MAE was less by 13.3%, RMSE was less by 14.1%, and R^2^ was higher by 2%; when compared to S2 and S3 results, S3’s MAE was less by 1.5%, RMSE was less by 1.7%, and R^2^ was higher by 0.2%. Overall, both in the training phase and the testing phase, the results were the same that S3 performed better results during temperature predictions.

As with IAT prediction, IRH prediction also followed the same results for the input datasets. For instance, RFR obtained best performance (with S3) (R^2^ = 0.9594; RMSE = 2.429; MAE = 1.470) during the IRH predictions. When compared to S3 and S1 results of RFR, S3’s MAE was less by 8.5%, RMSE was less by 7.96%, and R^2^ was higher by 0.7%; when compared to S2 and S3 results, S3’s MAE was less by 31.8%, RMSE was less by 27%, and R^2^ was higher by 2.6%. Likewise, the MLR obtained the least performance among other models during the IRH prediction (R^2^ = 0.780; RMSE = 2.429; MAE = 1.470). When compared to S3 and S1 results of MLP, S3’s MAE was less by 18.8%, RMSE was less by 16.5%, and R^2^ was higher by 10%; when compared to S2 and S3 results, S3’s MAE was less by 7.4%, RMSE was less by 7%, and R^2^ was higher by 4%. The complete results of prediction models along with different datasets and different phases during IAT and IRH predictions were shown in Table 4 and Table 5.

### 3.2. Model Performance

**Figure 6:**
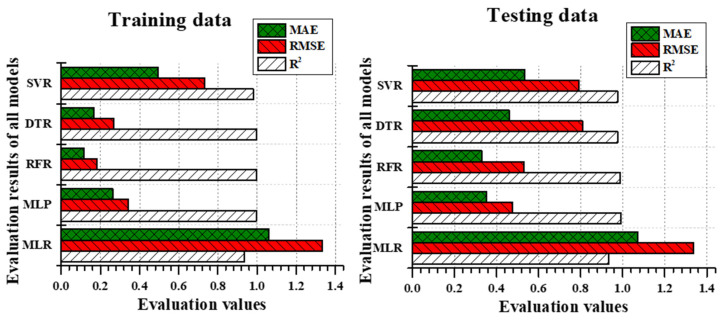
Training evaluation metric comparison between MLR, MLP, RFR, DTR, and SVR with S3 and testing evaluation metric comparison between those models during IAT prediction.

In IAT predictions, most of the models performed well during the training time in RMSE and R^2^. For instance, the results of all the models RMSE except MLR were less than 1 °C during the training phase, but the MLR model produces over 1 °C; similar results were obtained in MAE results. The training accuracy was high in the RFR model with S3 data than MLP, but in the testing phase, the results were vice versa. In terms of the percentage difference between RFR’s training and testing, results were 64.5% less in MAE, 65% less in RMSE, and 0.9% less in R^2^. However, MLP’s training and testing results were 25% less in MAE, 27% less in RMSE, and 0.4% less in R^2^. Interestingly, the MLR performed lower results than all other model outputs during training and testing, but the MLR’s training and testing results were 1% less in MAE, 0.2% less in RMSE, and 0.3% less R^2^. Though the differences between training and testing results were less in MLR, it performed significantly less accurate predictions than other models. The comparison of evaluation metrics between all the models during the training phase and testing phase is illustrated in Figure 6, where the MLP and RFR simulated similar results during the testing phase even though the training results between them were vice versa.

**Figure 7:**
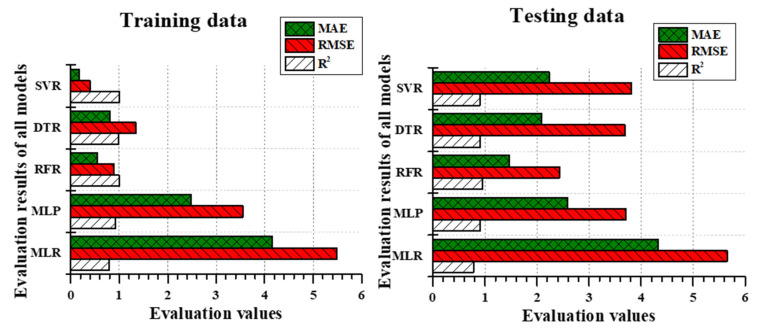
Training evaluation metric comparison between MLR, MLP, RFR, DTR, and SVR with S3 and training evaluation metric comparison between those models during IRH prediction.

In IRH predictions, the training results followed a similar pattern as IAT predictions. As like IAT training results, other models than MLR followed by MLP predicted IRH adequately. The RMSE results of RFR, DTR, and SVR were less than 1.5%, whereas MLP and MLR, respectively were 3.54 and 5.49, which were considerably high. Likewise, the MAE results were also high in MLR and MLP while in the training period. From the reference of Table 5, SVR performed better outcomes during the training phase, and the testing results were poor (R^2^ = 0.9; RMSE = 3.8161; MAE = 2.2302). Compared to the training and testing deviations of RFR, a considerable difference was noticed (92% high in MAE, 149% high in RMSE, and 10% less in R^2^). Even though the MLR and MLP performed poor outcomes, the difference between training and testing accuracy was not significant (MLR and MLP results followed by 4% and 4.15% was high in MAE; 2.8% and 4.3% was high in RMSE; 2.16% and 1% was low in R^2^). The compression of evaluation metrics between models are clearly illustrated in Figure 7.

Though the deviation between training and testing results was considerably high in RFR, the current study considered RFR model performance results during testing was satisfactory, among other models with the proof of Table 5 and Figure 7 (R^2^ = 0.9594; RMSE = 2.429; MAE = 1.47). The difference between training and testing accuracy for RFR was 62.6% in MAE, 63% in RMSE, and 3.6% in R^2^. Overall, RFR was considered a better model than DTR for IRH prediction.

### 3.3. Model Comparison

**Figure 8:**
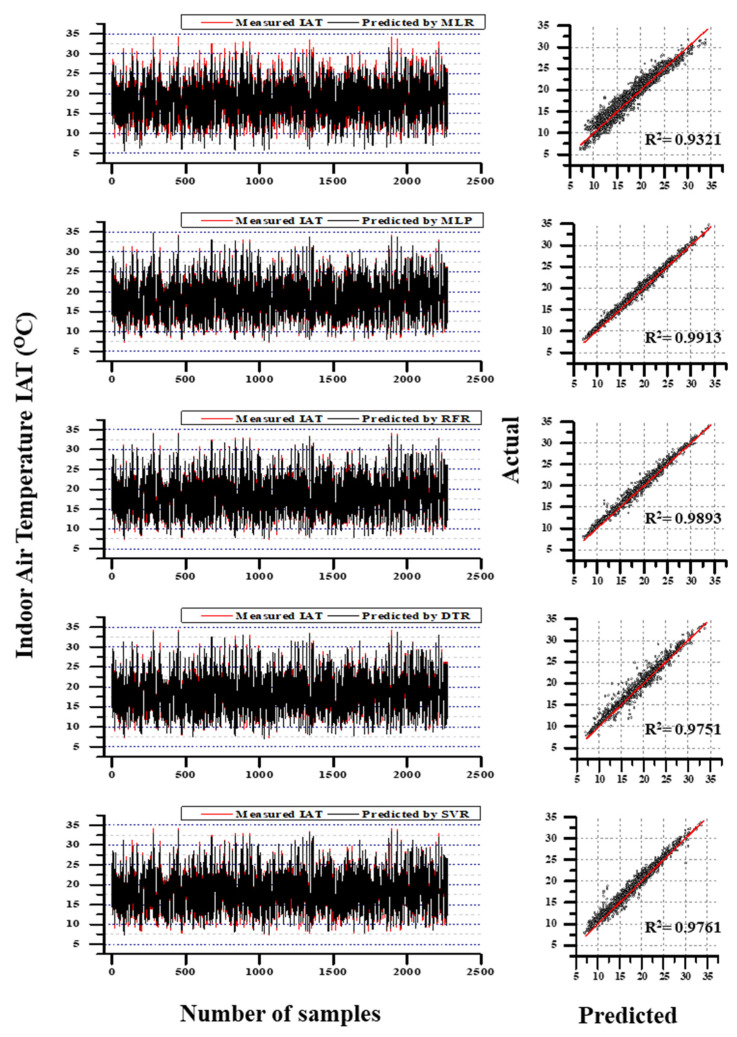
The comparison results between actual and simulated by MLR, MLP, RFR, DTR, and SVR with S3 for IAT prediction; the coefficient of determination between actual and predicted for all the models.

From the comparison results of IAT prediction, the MLP model performed better results during the testing phase. Since the training was supervised learning so that the testing results were treated as a substantial evaluation. Even though RFR’s training results and testing MAE were better than MLP. In training RFR results shows that MAE (55% low), RMSE (46.6% low) and R^2^ (0.3% higher); in testing, MAE (7% low). In terms of testing RMSE (10% lower) and R^2^ (0.2% higher) where MLP overcame RFR. Other than those models, SVR, DTR, and MLR performed 3rd, 4th, and 5th, respectively. When compared with MLP results, SVR was 50% higher in MAE, 66% higher in RMSE and 1.5% low in R^2^; DTR was 22% higher in MAE, 68% higher in RMSE, and 1.6% lower in R^2^; MLR was 203% higher in MAE, 180% higher in RMSE and 6% less in R^2^. The overall comparison between actual IAT values and predicted values along with the coefficient of determination values are illustrated in Figure 8.

**Figure 9:**
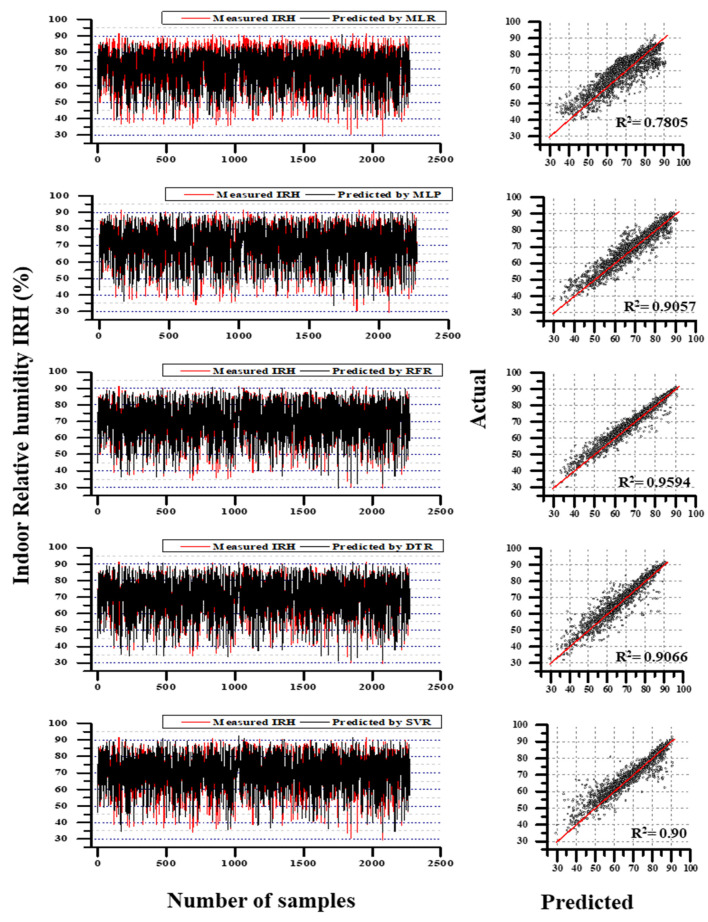
The comparison results between actual and simulated by MLR, MLP, RFR, DTR, and SVR with S3 for IRH prediction; the coefficient of determination between actual and predicted for all the models.

**Figure 10:**
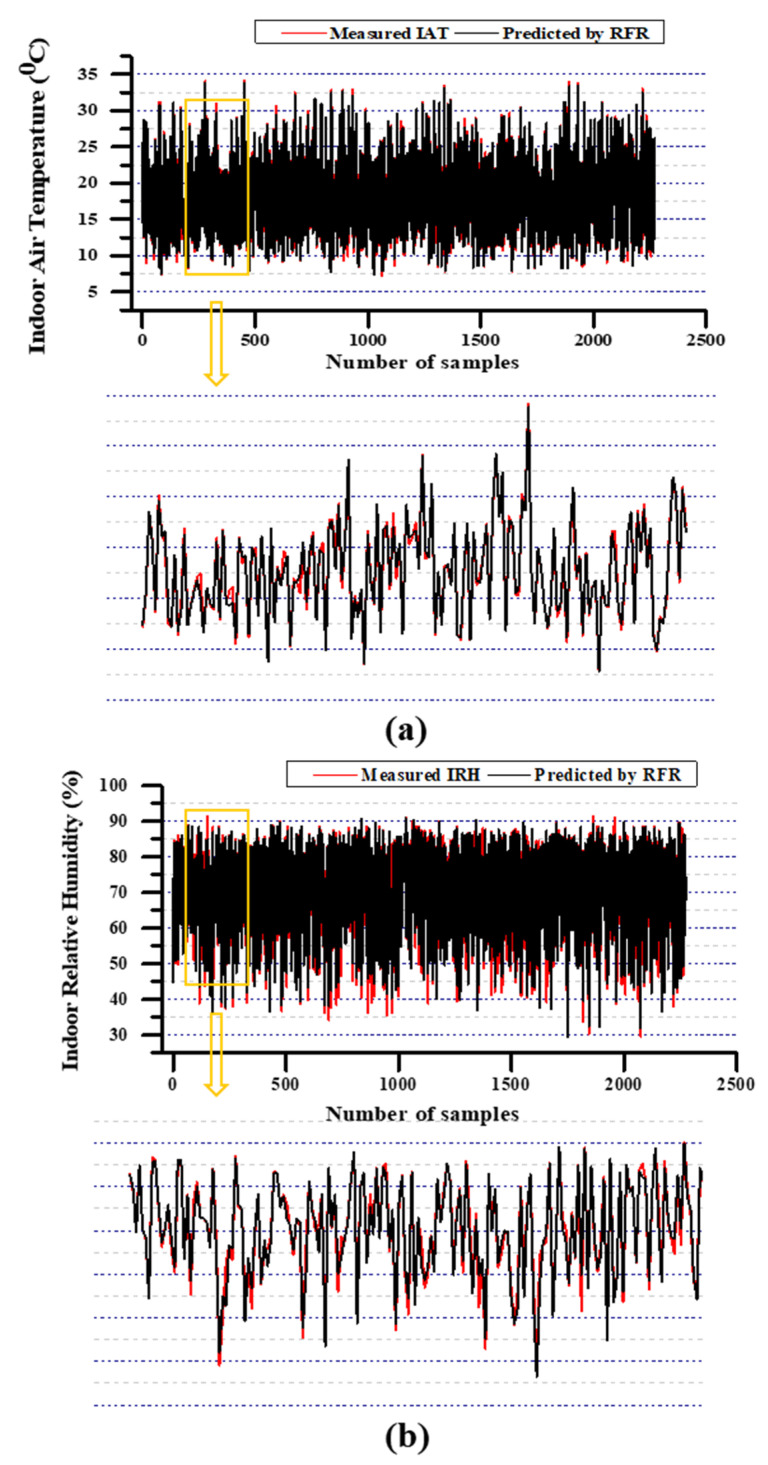
The comparison results between actual and simulated by RFR with S3 for (**a**) IAT prediction (**b**) IRH prediction including the zoomed view from the simulation results.

Likewise, IRH’s evaluation results (refer Table 5) illustrated that RFR performed better results (R^2^ = 0.9594; RMSE = 2.429; MAE = 1.4708) during the testing phase. Unlike IAT prediction performance, the models performed comparably less than the high-performance model. RFR, DTR, and SVR models produce better results during the training time, yet testing results are non-reliable except for RFR prediction. For instance, SVR’s training accuracy was better than RFR (MAE was 69.5% less, RMSE was 56% less, and R^2^ was 0.4% high); however, it was lagged to make reliable predictions using test data. When considering the R^2^ between SVR and RFR, 6% was still on a colossal scale to negate. Thus, all models except RFR have created a baffling circumstance to scale the stability. The performance of MLP models, which was considered the best performer in IAT predictions, was also turned to contradict during IRH predictions. The overall comparison between actual IRH values and predicted values along with the coefficient of determination values are illustrated in Figure 9. The comparison results between actual and simulated by RFR with S3 for IAT prediction IRH prediction including the zoomed view (randomly selected) from the simulation results are illustrated in Figure 10. However, according to the prediction result, DTR, MLP, SVR, MLR retained 2nd, 3rd, 4th, and 5th places, respectively. Compared to the RFR’s outcomes, DTR was 42% high in MAE, 51.8% high in RMSE, and 5.5% less in R^2^; MLP was 75.7% high in MAE, 52.5% high in RMSE, and 5.6% less in R^2^. The SVR was 51.6% high in MAE, 57% high in RMSE, and 6% low in R^2^; MLR was 194.5% high in MAE, 123% high in RMSE, and 17.8% low in R^2^. The aforementioned percentage differences were calculated from the high-performed model.

## 4. Discussion

### 4.1. Model Selection

Modeling is a commonly used mechanism for quantification of swine buildings’ microclimate [16,45,46]. The present study examined the popular ML models to predict IAT and IRH of a naturally ventilated swine building. According to our results, MLP performed better during IAT predictions, and RFR performed optimal during IRH prediction. Separate models can predict individual dependent variables, but IAT and IRH are parallel dependent variables of indoor microclimate, so those should predict together. Predicting these using two different models may require more time and computer usage. In addition, predicting dependent variables with single models is a straightforward and non-complex approach. A previous study [11] adopted four advanced ML models to predict the soil temperature (ST) in different depths, where the extreme learning machine (ELM) model outperformed in 5, 10, and 50 cm depths. Whereas in 100 cm depth, the MLR model performed better than the ELM models. That study compromised on the negligible amount of error metrics while considering the overall prediction performance and concluded that the ELM model is preferable for the ST predictions [11]. Therefore, when re-examining the results of this study, the RFR efficiency in the IRH predictions was exceptional indeed. Nevertheless, MLP and RFR simulated similar outcomes during IAT forecasts. Especially in terms of R^2^, 0.2% is a slight difference and could be negligible. Although MLP’s are considered optimal, their performance during IRH forecasts is not reliable. The performance of RFR and MLP has a non-consequential divergence during IAT predictions. Compared to MLP, RFR is versatile, fast during training, and is a less complex approach since it requires less parameter tuning. Considering the above criteria, the current research contemplates that RFR models are the optimal solution for predicting the IAT and IRT of a swine building.

As mentioned earlier, models other than MLR were trained and tested without optimization. The models were then optimized, and the appropriate hyper parameters were selected, re-trained, and tested. Both results were compared, and the best results were chosen as the best performance of the particular model. The current study observed all the utilized ML models performed better prediction after the optimization, which is concurring with previous literature [14,31,44]. Selecting the right hyper parameters has been demonstrated as the basic mechanism not only in the learning of ML models but also in obtaining optimum output utilizing the possible capability of the algorithms. Current research has observed that such over-fitting problems are solved when it is subjected to an optimization technique. Especially in DTR, the over fitting issue occurred more often if it was not optimized due to the nature of the algorithm. Choosing the right hyper parameter might lead to overcoming such training problems and producing a desirable prediction. All the experiments show that the mathematical model (MLR) is significantly lower than the ML models. Nevertheless, those are easy to handle and forthright, but it is limited to a scenario of extrapolating beyond the range of data. In such cases, computational models (ML) have an advantage over traditional statistical models since they could optimize according to data deviation.

### 4.2. Model Accomplishment

Providing optimal indoor environmental conditions provides optimum welfare and productivity in any livestock [7,12,46]. Pigs are highly sensitive to humidity more than temperature. However, relative humidity below 40% may contribute to excessive dustiness, which broadens the mortality rate of pigs [5,7]. Although many researchers have modelled, thermal conditions in the barn, temperature, and energy consumption, etc., research on humidity predictions are comparably scarce among livestock research. Current research has taken a substantial step toward addressing those deficiencies. For instance, [16] proposed an ARIMA based statistical model to predict the animal-zone temperature in weaned piglet buildings. The final finding of the indoor air temperature predictions was R^2^ = 0.134, which is comparably low than this study (R^2^ = 0.9913), though the RMSE of that literature was 0.204 (our proposed model = 0.476). However, the R^2^ and RMSE are the different characteristics to compare directly since those metrics are depend upon the data quality, amount, and deviation of the data. On the other hand, more attributes are given as input for any model increase the complexity of the model indeed. Previous literature used many complex predictor variables such as the volume of air extracted, power of ventilation system, the temperature of the heating plate, area of air outlet through the fan, live weight of the animal, and time of animal activity to predict IAT. Such parameters are difficult to collect; also, it requires more human resources. Even though the previous studies used such attributes, the R^2^ values prove that the current model could perform better results. In 2018, [17] used the same ARIMA model to predict animal zone temperature in a swine building, and the final R^2^ for the temperature prediction was in the range of 0.52–0.81, which is significantly less than the current proposed model. Both of the previous literature was simulated only the IAT of swine buildings, but the current study proposed both IAT and IRH predictions since both are essential to control.

In 2007, [3] was build a dynamic computer model for predicting indoor temperature and humidity of a pig barn. The R^2^ was 0.91 for IAT and 0.68 for IRH; besides, this literature validates the model only for two days. Previous author [45] designed a CFD based model to internal environmental conditions in a full-scale commercial pig house containing animals. Similar to the previous literature, [45] also used two days for model validation, and the RMSE was 5.52 for IAT and 17.5 for IRH. Model validation is imperative for indoor microclimate models to ensure the robustness and performance of the models; consequently, understand the reliability. When compared with our study, both the accuracy and validation was limited in the previous studies. Likewise, [10] proposed an ANN-based MLP model to predict the temperature and relative humidity of a swine building. The study validates the model by MSE and MAE; the IRH’s RMSE was better than our proposed model (RMSE = 0.8310), whereas the IAT’s RMSE was inferior (RMSE = 0.8095). Still, previous literature has no evidence of R^2^, which is an essential metric to evaluate the data extrapolation. Overall, the current study evaluated the performance between popular ML models and a statistical model during the prediction of IAT and IRH of a naturally ventilated swine building with three different input sets.

## 5. Conclusions and Application

Despite the advanced technologies at present, providing a comfortable environment for livestock is still considered a struggling phenomenon. Forecasting models are essential professionals for improving environmental control in livestock buildings. The current study successfully predicts IAT and IRH using simple and powerful ML models. In the end, this literature attempts to conclude with the following key points,

- The RFR models performed the most well among all the forecasting models used in this research most probably. RFR model has competent results in especially for IRH predictions compared with others. In addition, model-based control algorithms need to be developed for the real-time implementation of RFR based prediction integration in hardware.
- As seen in the results, the ML models used in this study have been more efficient than the statistical model. The statistical model was unable to make predictions when the data distribution is beyond the limit. Such models are limited to focus only on the linear relationship between variables. On the other hand, the ML models perform better with input variables that are complex and nonlinear due to the self-adaptive nature. ML models deem to be the optimal solver for the livestock indoor microclimatic control; since there are high fluctuations in the indoor environment of pig buildings and are very pervasive in general.
- The present study predicted IAT and IRH from accessible attributes without considering the animals’ biological factors. However, biological factors may affect the indoor climate still predictions of RFR have proven to simulate the parameters convincingly. Using accessible data rather than biological and non-accessible data can be better able to sustain human resources such as money, human needs, time, and technical resources such as computer usage, algorithm learning time, and model complexity.
- Selecting the right features from the given input data builds supportive conditions under which the expected results are available. Proving a greater number of attributes as input not only stifles the algorithm but also creates a confounding infrastructure to making the expected decisions. Witnessing the results of this study suggests that selecting the collect features is the most necessary process when modelling any indoor microclimate variables.
- The current study considered MLP, RFR, DTR, SVR, and MLR models to predict IAT and IRH. Recently deep learning (DL) and extreme learning machines (ELM) models are also enormously used to solve prediction problems. Such kind of models could be compared with the ML models in future studies. In addition, current literature used limited data due to the complication of collect indoor climate data for supervised learning. So in the future, big data for many cycles will be used to suggest an ultimate solution for controlling the indoor microclimate of swine buildings.

## Figures and Tables

**Table 1:** Descriptive statistics and profile information of the outdoor/predictor data collected from weather station (Campbell scientific weather station) and indoor/response data collected from LEMS sensors.

S. No	Attribute	Elements/Predictors (Unit)	Mean ± SD	SE	Min	Max
1	WD	Wind direction (°(Azimuth))	205.0 ± 67.43	0.632	29.4	337.2
2	WS	Wind speed (m/s)	0.644 ± 0.379	0.003	0.11	4.55
3	OAT	Outdoor air temperature (°C)	12.858 ± 6.729	0.063	−2.7	31
4	ORH	Outdoor relative humidity (%)	72.746 ± 22.082	0.207	13.78	96.9
5	AP	Outdoor air pressure (Pa)	1013.916 ± 5.495	0.051	976	1024
6	RFA	Rain fall amount (inch)	0.0057 ± 0.059	0.0005	0	1.71
7	SLR	Solar irradiance (Wm-2)	124.722 ± 199.280	1.869	0	889
8	SMC	Soil moisture content (%)	17.325 ± 1.722	0.016	13.88	29.62
9	ST	Outdoor soil temperature (°C)	13.851 ± 6.229	0.058	2.622	30.26
10	CNR	Net radiation (Wm-2)	31.037 ± 149.867	1.406	−161.8	645.1
**S. No**	**Attribute**	**Elements/Response (Unit)**	**Mean ± SD**	**SE**	**Min**	**Max**
1	IAT	Indoor air temperature (°C)	18.294 ± 5.22	0.048	6.7	34.2
2	IRH	Indoor relative humidity (%)	70.122 ± 12.179	0.114	25.5	92.3

**Table 2:** Summary of the attributes which were chosen to train the model named S1, S2, and S3 during IAT and IRH predictions.

Model	Datasets	Description	Response
S1	WD, WS, OAT, ORH, AP, RFA, SLR, SW, ST, CNR	All Collected parameters from weather station	IAT
S2	WD, WS, OAT, ORH, AP, RFA, SLR, SW, ST, CNR, IRH	All Collected parameters from weather station including indoor parameters	IAT
S3	OAT, ORH, ST, SLR, IRH	Selected feature by using correlation matrix (Including positive and negative relationship by using Spearman rank correlation coefficient approach)	IAT
S1	WD, WS, OAT, ORH, AP, RFA, SLR, SW, ST, CNR	All Collected parameters from weather station	IRH
S2	WD, WS, OAT, ORH, AP, RFA, SLR, SW, ST, CNR, IAT	All Collected parameters from weather station including indoor parameters	IRH
S3	ORH, SLR, CNR IAT	Selected feature by using correlation matrix (Including positive and negative relationship by using Spearman rank correlation coefficient approach)	IRH

**Table 3:** The range of critical hyper parameters tuned during the prediction.

Algorithms	Hyper Parameters	Distribution (Range)
Multiple linear regression (MLR)	-	-
Multilayered perceptron (MLP)	Number of Hidden layers	*U_d_ (1, 4)
	Number of Hidden neurons	U_d_ (1, 250)
	Learning rate	Adaptive
	Solver	Adam
	Activation function	Relu
Decision tree regression (DTR)	Maximum depth	U_d_ (1, 100)
	Minimum sample split	U_d_ (2, 10)
	Minimum sample leaf	U_d_ (1, 4)
Support vector regression (SVR)	Kernel	Radial-basis function
	C	U_d_ (1, 100)
	Gamma	1
	Epsilon	0.1
Random forest regression (RFR)	Number of trees	U_d_ (10, 250)
	Minimum number of observations in a leaf	U_d_ (1, 30)
	Number of variables used in each split	U_d_ (1, 4)
	Maximum tree depth	U_d_ (1, 100)

* U_d_ stands for uniform discrete random distribution from a to b.

**Table 4:** The performance assessment of all the models along with S1, S2, and S3 input data set during IAT predictions.

S1						
Models	Training			Validation		
	MAE *	RMSE *	R^2^ *	MAE	RMSE	R^2^
MLR	1.2022	1.5202	0.9159	1.2254	1.558	0.9076
**MLP**	**0.2832**	**0.3808**	**0.9947**	**0.3719**	**0.5301**	**0.9893**
**RFR**	**0.1271**	**0.2088**	**0.9984**	**0.3574**	**0.5807**	**0.9871**
DTR	0.1939	0.3351	0.9959	0.4979	0.899	0.9692
SVR	0.6731	0.9865	0.9645	0.7302	1.0878	0.9549
**S2**						
**Models**	**Training**			**Validation**		
	**MAE**	**RMSE**	**R^2^**	**MAE**	**RMSE**	**R^2^**
MLR	1.0772	1.3557	0.9331	1.087	1.3551	0.9301
**MLP**	**0.3968**	**0.54**	**0.9893**	**0.4459**	**0.621**	**0.9853**
**RFR**	**0.126**	**0.2013**	**0.9985**	**0.3641**	**0.5903**	**0.9867**
DTR	0.1933	0.3194	0.9962	0.5003	0.8539	0.9722
SVR	0.5846	0.8204	0.9755	0.6097	0.8613	0.9717
**S3**						
**Models**	**Training**			**Validation**		
	**MAE**	**RMSE**	**R^2^**	**MAE**	**RMSE**	**R^2^**
MLR	1.061	1.332	0.9354	1.0721	1.3352	0.9321
**MLP**	**0.2628**	**0.3434**	**0.9957**	**0.3535**	**0.4763**	**0.9913**
**RFR**	**0.1165**	**0.1833**	**0.9987**	**0.3282**	**0.5283**	**0.9893**
DTR	0.1648	0.2683	0.9973	0.4595	0.8081	0.9751
SVR	0.4936	0.7333	0.9804	0.5331	0.7911	0.9761

* MAE—Mean absolute error; RMSE—Root mean square error, R^2^—coefficient of determination; Bold fonts represents top performed results with the corresponding data set.

**Table 5:** The performance assessment of all the models along with S1, S2, and S3 input data set during IRH predictions.

S1						
Models	Training			Validation		
	MAE	RMSE	R^2^	MAE	RMSE	R^2^
MLR	4.9058	6.2697	0.7361	5.1502	6.589	0.7019
MLP	3.3399	4.503	0.8638	3.5312	4.7917	0.8423
**RFR**	**0.5931**	**0.9607**	**0.9938**	**1.5963**	**2.6222**	**0.9527**
**DTR**	**0.8047**	**1.3866**	**0.987**	**2.0807**	**3.5936**	**0.9113**
SVR	0.2385	0.6668	0.997	2.4453	4.028	0.8886
**S2**						
**Models**	**Training**			**Validation**		
	**MAE**	**RMSE**	**R^2^**	**MAE**	**RMSE**	**R^2^**
MLR	4.4651	5.8377	0.7712	4.6572	6.0534	0.7484
MLP	3.238	4.4669	0.866	3.4088	4.7072	0.8478
**RFR**	**0.7206**	**1.1475**	**0.9911**	**1.9392**	**3.0872**	**0.9345**
DTR	1.6783	2.635	0.9533	2.6972	4.3607	0.8694
**SVR**	**0.8696**	**1.1269**	**0.9914**	**2.1301**	**3.4244**	**0.9194**
**S3**						
**Models**	**Training**			**Validation**		
	**MAE**	**RMSE**	**R^2^**	**MAE**	**RMSE**	**R^2^**
MLR	4.1603	5.4935	0.7974	4.3323	5.653	0.78058
MLP	2.4782	3.5452	0.9156	2.5856	3.7046	0.9057
**RFR**	**0.5494**	**0.8847**	**0.9947**	**1.4708**	**2.429**	**0.9594**
**DTR**	**0.7985**	**1.3353**	**0.988**	**2.0876**	**3.6876**	**0.9066**
SVR	0.1671	0.3896	0.9989	2.2302	3.8161	0.9

## Data Availability

No new data were created or analyzed in this study. Data sharing is not applicable to this article.
